# Supplementary material for: Comprehensive Annotation and Functional Exploration of MicroRNAs in Lettuce
Source: Front Plant Sci. 2021 Dec 24;12:781836. doi: 10.3389/fpls.2021.781836 (PMC8739914; doi:10.3389/fpls.2021.781836)
Supplement: Supplementary file 3 [file Data_Sheet_9.ZIP › GSM2230752.plot/Lsa-miRN48_Lsat_1_v5_gn_0_37821.1_327_TPlot.pdf]

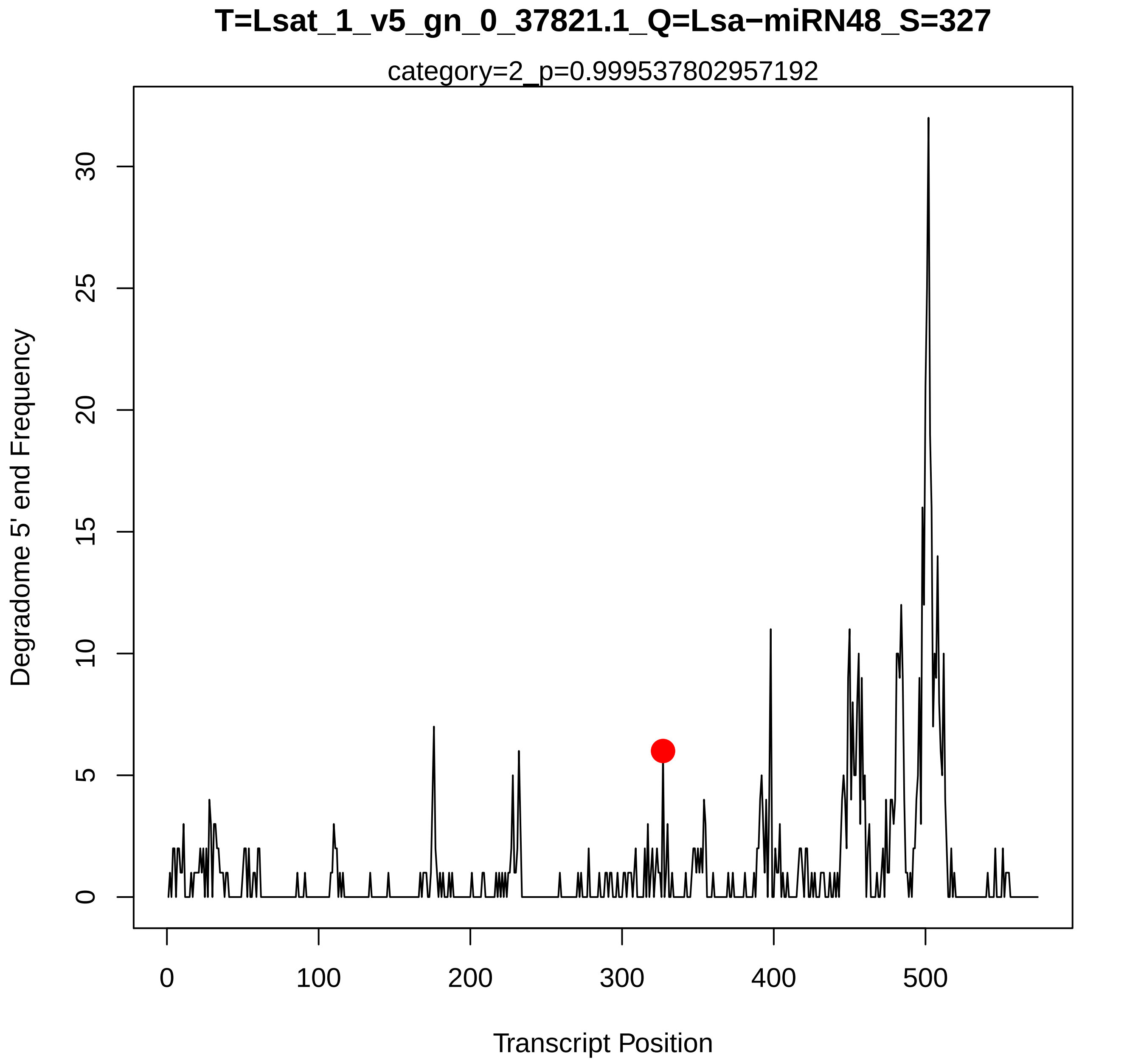

T=Lsat_1_v5_gn_0_37821.1_Q=Lsa−miRN48_S=327
category=2_p=0.999537802957192
Degradome 5' end Frequency
Transcript Position
0
5
10
15
20
25
30
0
100
200
300
400
500
